# Febrile attacks triggered by milk allergy in an infant with mevalonate kinase deficiency

**DOI:** 10.1007/s00296-016-3522-3

**Published:** 2016-07-07

**Authors:** Hideyuki Nakashimai, Fumihito Miyake, Shigeru Ohki, Seira Hattori, Tadashi Matsubayashi, Kazushi Izawa, Ryuta Nishikomori, Toshio Heike, Yoshitaka Honda, Yosuke Shigematsu

**Affiliations:** 1 Department of Neonatology, Seirei Hamamatsu General Hospital, 2-12-12 Sumiyoshi, Naka-ku, Hamamatsu, Shizuoka 430-8558 Japan; 2 Department of Pediatrics, Seirei Hamamatsu General Hospital, Hamamatsu, Shizuoka Japan; 3 Department of Pediatrics, Graduate School of Medicine, Kyoto University, Kyoto, Japan; 4 Department of Health Science, Faculty of Medical Sciences, University of Fukui, Fukui, Japan

To the Editor,

Mevalonate kinase deficiency (MKD) is a rare autoinflammatory disease characterized by recurrent fever, lymphadenopathy, hepatomegaly, splenomegaly, skin lesions, abdominal pain, and diarrhea [1–4] and may lead to neurological involvement and death [1, 5]. The febrile attack is commonly triggered by infection, vaccines, and stress. We report an infant with MKD whose febrile attacks were triggered by milk allergy.

The female patient was born by cesarean section because of obstructed labor at a gestational age of 39 weeks and 4 days with a birth weight of 2612 g. No signs of infection were seen in the gestation and peripartum period. Her father was Hispanic and her mother Asian. At 3 days of age, her body temperature rose above 38 °C, and the next day she was transferred to the neonatal intensive care unit of Seirei Hamamatsu General Hospital, Shizuoka, Japan. On admission, the patient’s activity was maintained, and she could feed breast and formula milk adequately. Body temperature was 39.0 °C and mild tachypnea was observed. No aphthous ulcers in the oral cavity were observed. Breath sounds were normal and heart murmurs were not audible. The abdomen was slightly distended but hepatosplenomegaly was not observed. Laboratory data showed: white blood cell count 15,010 /μL, red blood cell count 379 × 10^4^ /μL, hemoglobin 12.4 g/dL, hematocrit 36.7 %, platelet count 30.3 × 10^4^ /μL, total protein 6.4 g/dL, albumin 3.0 g/dL, total bilirubin 2.5 mg/dL, aspartate aminotransferase 112 IU/L, alanine aminotransferase 67 IU/L, lactate dehydrogenase 660 IU/L, creatine phosphokinase 63 IU/L, blood urea nitrogen 5 mg/dL, creatinine 0.30 mg/dL, C-reactive protein 13.3 mg/dL, immunoglobulin (Ig) G 945 mg/dL, IgA 3 mg/dL and IgM 91 mg/dL. Neither pyuria nor pleocytosis were present. Ophthalmological examination confirmed that there was no uveitis. At 6 days old, small red papules appeared on the skin of the patient’s entire body. No significant pathogens were cultured from her urine, blood, stool, spinal fluid or pharyngeal secretion and no virus was isolated from her stool and pharyngeal secretion.

Antibacterial and antiviral agents were infused but the fever persisted. At the age of 16 days, the patient became sluggish and abdominal distension increased with watery diarrhea. C-reactive protein markedly elevated to 41.2 mg/dL. Computed tomography scan showed dilatation of the intestine, edematous intestinal walls and retention of the intestinal contents. Serum IgE increased to 367.8 IU/mL and IgE specific for cow’s milk to 4.07 IU/mL. Both human and formula milk were ceased. The fever declined gradually, she became active, and the abdominal distension and diarrhea improved. Human milk and elemental diet were tolerated, but re-introduction of formula milk induced fever and watery diarrhea again. Urine concentrations of mevalonic acid in the afebrile and febrile periods were elevated to 26.5 and 35.9 µg/mg Cr, respectively. Mevalonate kinase activity with respect to control values was 1.3 % and *Mevalonate kinase* gene analysis revealed a compound heterozygous mutation [c.613A > G/c.382_383 del AG].

In MKD, the inflammasome, which converts pro-IL1β to IL1β and causes fever, is activated excessively by exogenous and endogenous microbial products [4–6]. The disease is caused by the lack of isoprenoids (mevalonate pathway products), and mevalonic aciduria is characteristic. The inflammatory responses in this case were too strong to be induced by milk allergy alone, thus we predicted the co-existence of another inflammatory disease. Although the mechanism is not precisely understood, tissue damage by milk allergy may activate the inflammasome.

At 2 months of age, febrile attack can be avoided by disuse of formula milk. Later, however, attacks may be induced by things such as initiation of vaccinations and common colds. If attacks occur repeatedly, anti-inflammatory drugs such as IL-1 blockers should be introduced to prevent severe complications.
